# Conformer‐RL: A deep reinforcement learning library for conformer generation

**DOI:** 10.1002/jcc.26984

**Published:** 2022-08-24

**Authors:** Runxuan Jiang, Tarun Gogineni, Joshua Kammeraad, Yifei He, Ambuj Tewari, Paul M. Zimmerman

**Affiliations:** ^1^ Department of EECS University of Michigan Ann Arbor Michigan USA; ^2^ Department of Statistics University of Michigan Ann Arbor Michigan USA; ^3^ Department of Chemistry University of Michigan Ann Arbor Michigan USA

**Keywords:** conformer generation, graph neural network, machine learning, reinforcement learning

## Abstract

Conformer‐RL is an open‐source Python package for applying deep reinforcement learning (RL) to the task of generating a diverse set of low‐energy conformations for a single molecule. The library features a simple interface to train a deep RL conformer generation model on any covalently bonded molecule or polymer, including most drug‐like molecules. Under the hood, it implements state‐of‐the‐art RL algorithms and graph neural network architectures tuned specifically for molecular structures. Conformer‐RL is also a platform for researching new algorithms and neural network architectures for conformer generation, as the library contains modular class interfaces for RL environments and agents, allowing users to easily swap components with their own implementations. Additionally, it comes with tools to visualize and save generated conformers for further analysis. Conformer‐RL is well‐tested and thoroughly documented with tutorials for each of the functionalities mentioned above, and is available on PyPi and Github: https://github.com/ZimmermanGroup/conformer-rl.

## Graphical abstract

Conformer‐RL is an open‐source Python package for generating conformers of molecules and polymers using deep reinforcement learning. The package includes pretrained models for generating conformers of several classes of covalently bonded molecules as well as a robust library for training and evaluating tailored models for custom molecules and tasks.

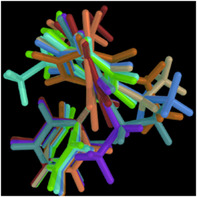

## 1 INTRODUCTION

Reinforcement learning (RL) is a machine learning technique where an intelligent agent is trained by being given a “reward” or “penalty” based on the outcome of its actions. Historically, these methods have seen great success in strategy games like chess^1^ and StarCraft II.^2^ Several recent works have applied deep RL to tasks in computational chemistry as well.^3^, ^4^, ^5^ One task where deep RL has shown promising results is conformer generation, which involves finding an ensemble of unique low‐energy three‐dimensional orientations, or conformers, for a given molecule.^6^ Efficient and accurate prediction of low‐energy conformers is integral to molecular modeling, with wide applications from drug development to 3D QSAR.^7^

Since molecule conformations are determined by the rotation of their single bonds, the number of possible conformations grows exponentially as the number of bonds increases. This situation makes it difficult to come up with efficient algorithms for conformer generation. For example, existing advanced chemoinformatic methods for conformer generation, such as molecular dynamics (MD), or the enhanced self‐guided molecular dynamics (SGMD)^8^ simulations, which uses a gradient descent method with momentum and adaptive bias to find several local minimums in the energy surface of the conformer space, can take several days to sample conformers with 20+ rotatable bonds.^9^

Gogineni et al.^9^ found that defining the task of conformer generation as a RL problem leads to models that can generate a diverse set of conformers more efficiently than methods like MD and other machine learning methods like generative algorithms. To evaluate the conformers, the study used a metric that incorporates both the energy of each conformer and the diversity across the generated conformers (this metric is also implemented in Conformer‐RL as the “Boltzmann Factor Reward” in section RL Environments). Using this metric, the study found that a trained RL agent is highly effective, even when compared to specialized sampling methods like SGMD. On the task of generating conformers for a 8‐monomer lignin molecule, the trained RL model consistently performed better than SGMD (and even better than MD, in terms of finding low‐energy conformers), while sampling 10x fewer conformers and requiring less than 1% of the cpu runtime.

Nevertheless, building and training these models from scratch can be difficult and time consuming. While libraries already exist that contain implementations of RL algorithms and benchmarking tasks, such as RLlib^10^ and OpenAI gym,^11^ respectively, these packages do not work out of the box with chemical applications and require significant modification and programming knowledge to work with molecule structures.

In this paper, we introduce Conformer‐RL, a comprehensive and modular Python library for applying deep RL to conformer generation and other related tasks, using PyTorch^12^ for deep learning and RDKit^13^ for chemoinformatic capabilities. Conformer‐RL provides a set of tools to train models for generating conformers without the need for extensive knowledge of RL and programming. It includes a simple interface where users can train and save an RL agent given only a molecule file and configurable options for hyperparameters as input. A sequence of molecule files can also be used as input to train a model that can better generalize to a specific class of molecules (see Curriculum Learning). Currently, Conformer‐RL works with any covalently bonded molecule, though torsions within rings will be considered rigid when sampling conformers. The software will then output the trained model, which can generate conformers for the same or structurally similar molecules as the one used in training. When generating conformers using the trained model, Conformer‐RL will output the .mol files for each of the generated conformers, which can be used for further downstream tasks.

Due to the modular nature of Conformer‐RL's source code, it is also a framework where custom agents, training algorithms, neural networks, and other model components can be built and evaluated for conformer generation and similar tasks. As the state‐of‐the‐art techniques in deep RL are changing rapidly, this framework makes it easy to develop and test new RL ideas on this specific task. Specifically, Conformer‐RL includes a modular class to easily build interfaces for custom RL tasks for further exploration within conformer generation and for custom tasks like reaction prediction. Within Conformer‐RL, we include a general agent base class for building agents compatible with conformer generation tasks, as well as several baseline RL algorithms. Conformer‐RL provides analysis and logging modules for recording and visualizing training results, including conformer‐generation specific metrics and visuals.

This software is open‐source and free of charge for all users. The source code for the library can be found at https://github.com/ZimmermanGroup/conformer-rl, and installation instructions and full documentation can be found at https://conformer-rl.readthedocs.io/en/latest/. The project is maintained by graduate students in the Tewari and Zimmerman groups at the University of Michigan, who will provide support for external users and continue to build upon the platform described here. For contributing to Conformer‐RL and for feature requests or bug reports, please see the developer documentation at https://conformer-rl.readthedocs.io/en/latest/developer.html.

## 2 METHODS

**FIGURE 1:**
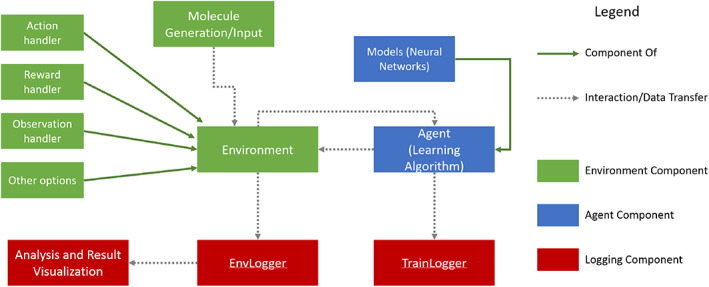
Architecture of Conformer‐RL

In this section, we discuss how Conformer‐RL frames conformer generation as a RL problem, as well as the implementation details for each component of the training framework. The architecture of Conformer‐RL is shown in Figure 1.

### 2.1 Reinforcement learning

A RL system involves an “RL environment,” which is the software interface for simulating the task, as well as an “agent,” which learns by interacting with the RL environment. Before any interaction, the RL environment will have some starting “state,” which we denote as *s*_0_. The agent interacts with the RL environment by sequentially selecting an “action” on the environment, which we will denote as *a*_*i*_, representing the *i*th action performed so far. After each action, the RL environment will update its internal state based on the action, leading to a new state *s*_*i*_. The RL environment will also calculate a “reward” *r*_*i*_ depending on the action and the previous state. The RL environment will then send the new state and reward (*s*_*i*_,*r*_*i*_) back to the agent, which the agent will use to determine the next action to take *a*_*i*+1_. The goal of the agent is to maximize the total reward achieved.

#### 2.1.1 RL for conformer generation

Conformer‐RL makes several assumptions when converting the conformer generation task into a RL problem. First, we assume that the bond lengths and bond angles are constant across conformers, so that each conformer is only determined by its torsion angles. This same assumption is used by other conformer generation algorithms, such as Confab.^14^ We further assume that all ring torsions are rigid, so we only sample torsion angles from non‐ring torsions. Finally, we assume that for each non‐ring torsion angle, all low‐energy conformers with different angles for that torsion will have a difference in angle of at least 60° for that same torsion. Using this assumption, we can sample each torsion angle from discrete “buckets” of 60°, instead of a continuous range of angles. The angle variations can be easily changed if needed, and future work will address sampling of ring conformers as well.

**FIGURE 2:**
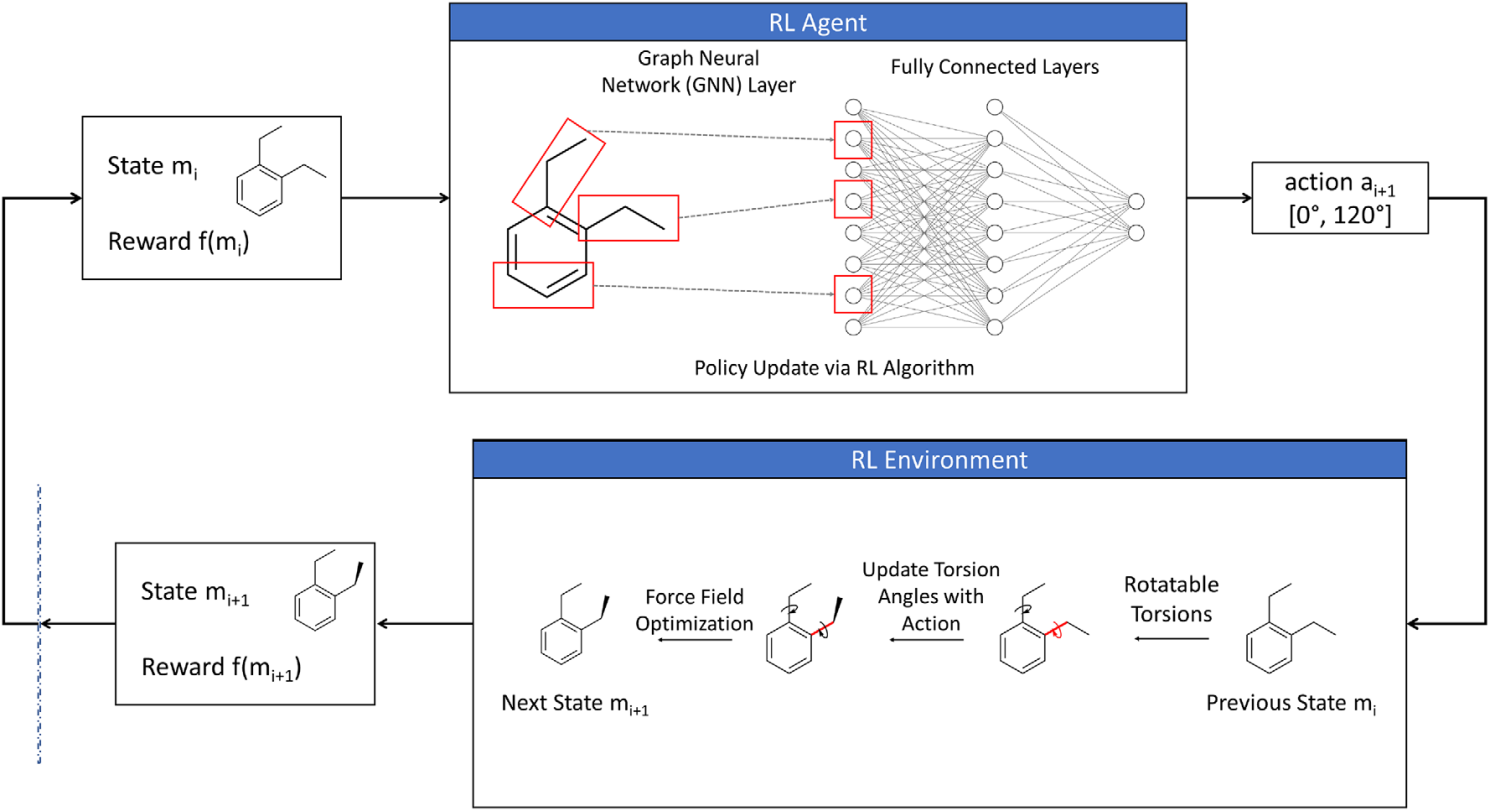
Illustration of an agent interacting with the reinforcement learning environment in a conformer generation task for a simple molecule with two torsions. At iteration *i*, the environment state is the conformation of the molecule with each torsion at 0°. After the agent interacts with environment with the action [0,120], the environment sets the first torsion angle to 0° and the second torsion angle to 120°. The conformer is optimized using a molecular force field to get to the conformer state for the next iteration, where generally the angles will not be exactly equal to the action‐specified angles.

We now describe the full details for the RL system (Figure 2). Suppose that we want to generate *C* conformers for a molecule, which has *n* rotatable torsions. We will number the torsions as [*t*_1_,*t*_2_,…*t*_*n*_] where *t*_*j*_ represents the *j*th torsion. The state of the RL environment corresponds to a conformation of the molecule. The initial state of the RL environment is a random conformer for the molecule whose conformer structure has been optimized using a molecular force field. We will denote this initial state as *m*_0_. The reward function, which we will denote as *f*, depends on the energy of the current molecule conformation, and in this case *f*(*m*_0_) is the reward associated with the initial state/conformer. Conformer‐RL implements several different reward functions, which are detailed in the RL Environments section. In the *i*th iteration of the RL environment, the agent is first given the current state and reward (*m*_*i*_,*f*[*m*_*i*_]). The agent outputs the next action *a*_*i*+1_, which is a vector of length *n*, where each element of the vector is a multiple of 60 within the interval [0,360]. We can write *a*_*i*+1_ as [*a*_*i*+1,1_,*a*_*i*+1,2_,…,*a*_*i*+1,*n*_]. Given this action, the RL environment will generate a new conformer by setting the torsion angle of torsion *t*_*j*_ to *a*_*i*+1,*j*_ for all 1 ≤ *j* ≤ *n*. Then, the resulting conformer structure is further optimized using a force field, and the resulting conformer will be the next state *m*_*i*+1_, and the corresponding reward will be *f*(*m*_*i*+1_). This cycle repeats until *C* cycles are completed, after which exactly *C* conformers will have been generated. A diagram of a single iteration of interaction between the agent and RL environment is shown in Figure 2.

Potential use cases for Conformer‐RL include a variety of types of organic molecules including most drug‐like molecules, and also includes linear or branched organic polymers. Molecules may contain rings but the torsions within these rings are not currently sampled. Potential future extensions include explicit sampling of ring torsions and sampling of intermolecular interactions between non‐covalently bonded molecular species.

#### 2.1.2 RL environments

A conformer generation RL environment can be created with any covalently bonded molecule as input, and also includes configurable options. The molecular structure is specified as an RDKit^13^ molecule object. As a mature cheminformatics library, RDKit offers a standard means of representing and manipulating molecules, and can interface across different formats from a number of computational chemistry packages including the MOL file format. RDKit is also at the core of a broad ecosystem of other cheminformatics packages, including Open Babel, which has extensive conversion capabilities for over 100 formats.^15^

RL environments are initialized with a MolConfig object, which specifies the RDKit molecule to be used in the RL environment and any molecule‐specific parameters. For convenience, Conformer‐RL contains scripts for generating MolConfig objects for several classes of molecules and polymers with conformer generation benchmarks found in Gogineni et al.,^9^ such as branched alkanes and lignin polymers. Molecule generation scripts utilize several libraries depending on the molecule, including stk,^16^ stko^17^ and Lignin‐KMC,^18^ with options for varying molecule size and structure. The library also includes convenience functions to automatically convert a molecule in a MOL file into a MolConfig object.

Conformer‐RL includes several configurable options for each component of the RL environment. Due to the flexibility of the design, different component implementations can be mixed and matched depending on the user's specific task, and new RL environments for tasks related to conformer generation, such as protein folding and chemical reaction optimization,^19^ can be easily built by implementing custom variations of the components. The main components include:

- *Action Handler* determines how the molecular structure is modified given an incoming action. The implementation discussed above, where the action is a list of integers that are multiples of 60, is included. Other implementations include torsion angle “buckets” of finer granularity than 60 degrees, as well as the option for the angle to be set to any real number in the continuous range [0,360]. After the torsion angles of the molecule are set, the conformer structure is further optimized using the MMFF94^20^ force field.
- *Reward Handler* specifies the function for the reward based on the current molecule conformation. Conformer‐RL includes several reward implementations derived from the energy of the current molecule conformation, such as (but not limited to):
  - *Basic Energy Reward*—reward that is inversely correlated with the energy of the current conformer. Formally,
$$f\left(m\right)=-E\left(m\right)$$
  where *f* is the reward function, *m* is the molecule conformer, and *E*(*m*) is the energy of the conformer.
  - *Pruning Energy Reward*—reward that “prunes” (returns a 0 reward) any conformer generated from an action already seen in the current episode.
  - *Boltzmann Factor Reward*—uses the Boltzmann factor of the conformer as the reward, and prunes conformers that are too similar to previously generated conformers using a torsional fingerprint distance (TFD)^21^ metric. Formally,
$$f\left(m\right)=\left\{\begin{array}{ll} 0 & \min\left\{\mathrm{TFD}\left(m,x\right):x\in S\right\}<\text{threshold}\\ e^{\frac{-\left(E\left(m\right)-E_{0}\right)}{\mathrm{kT}}} & \text{otherwise} \end{array}\right.$$

where *f* is the reward function, *m* is the conformer of interest, *S* is the set of all previously generated conformers, *E*(*m*) is the energy of *m*, *E*_0_ is a normalizing factor, and *TFD*(*m*,*x*) is the torsional fingerprint distance between conformers *m* and *x*.

- *Observation Handler* returns a graph representation for the current molecule conformation and specifies what features of the molecule conformation will be sent to the agent. Conformer‐RL contains several methods for extracting features from molecules and converting a conformation into a PyTorch Geometric graph structure, such as:
  - *Node Feature Extractors*—extracts information about atoms in a molecule which can be included in the nodes of the graph representation, such as atom element and three‐dimensional coordinates.
  - *Edge Feature Extractors*—extracts information from molecules that can be represented as edges in the graph reperesentation, with options for including bonds between atoms, bond type, Euclidean distances, and more.
  - *Graph Normalizers*—normalizes the graph representation of molecules in terms of translation, rotation, and/or scaling.

Conformer‐RL also includes options for executing multiple environments in parallel for faster performance on systems with multiple CPU cores.

#### 2.1.3 Agents and models

In deep RL, agents are trained on an RL environment by an RL algorithm, which learns from the RL environment by interacting with it and receiving feedback (in the form of a reward). Using this interaction, the agent develops a policy, or strategy for choosing actions, that leads to higher rewards. Conformer‐RL implements several state‐of‐the‐art RL algorithms using deep neural networks that can be used to train agents on any of the conformer generation RL environments described above. The RL algorithms include advantage actor critic (A2C)^22^ and proximal policy optimization (PPO).^23^ Both algorithms are policy gradient algorithms, which search for better policies by estimating the gradient of the total reward with respect to the policy. Both algorithms have been shown to perform well on the conformer generation task.^9^ The software also includes implementations of several modern graph neural network architectures modified to be compatible with molecular inputs, including versions of the model from the work of Gogineni et al.,^9^ which are used by the agent to learn the RL environment. The networks are built using PyTorch Geometric^24^ and are compatible with molecules of variable size.

### 2.2 Curriculum learning

Curriculum learning is a machine learning technique similar to transfer learning, where a model is trained on easier tasks initially, and then gradually more difficult tasks when the model has started to learn the current task. Recent empirical results have shown that curriculum learning significantly improves agent performance of RL agents in game tasks like Ms. Pac‐Man.^25^ Although transfer learning, which involves reusing a model trained on a task on a different task, has been used for chemistry applications like drug discovery,^26^ the use of curriculum learning is not widely explored.

Conformer‐RL allows users to utilize curriculum learning when training agents, simply by inputting a list of molecules when creating the RL environment rather than a single molecule. The agent will be first trained on the first molecule in the list, and then sequentially move to consecutive molecules once a performance threshold is reached. An example of curriculum learning is discussed in the section Example Usage.

### 2.3 Model selection and evaluation

Conformer‐RL contains tools for monitoring training progress and evaluating trained models, to aid in the selection of model hyperparameters. During training, the software's TrainLogger module logs information from the agent, such as total reward per episode, training loss, runtime, etc., and supports logging data directly to TensorBoard,^27^ where the data can be visualized in real time. To assess the generalization capabilities of the model during training, the system can also take a second RL environment as input. The model will not directly train on the second RL environment, but in every set number of training iterations, the model will be evaluated on the second RL environment. This can be useful for determining whether the trained model is able to generalize to other molecules besides the one it is training on, and which training iteration yields the best model on the evaluation RL environment.

### 2.4 Molecule visualization and analysis

**FIGURE 3:**
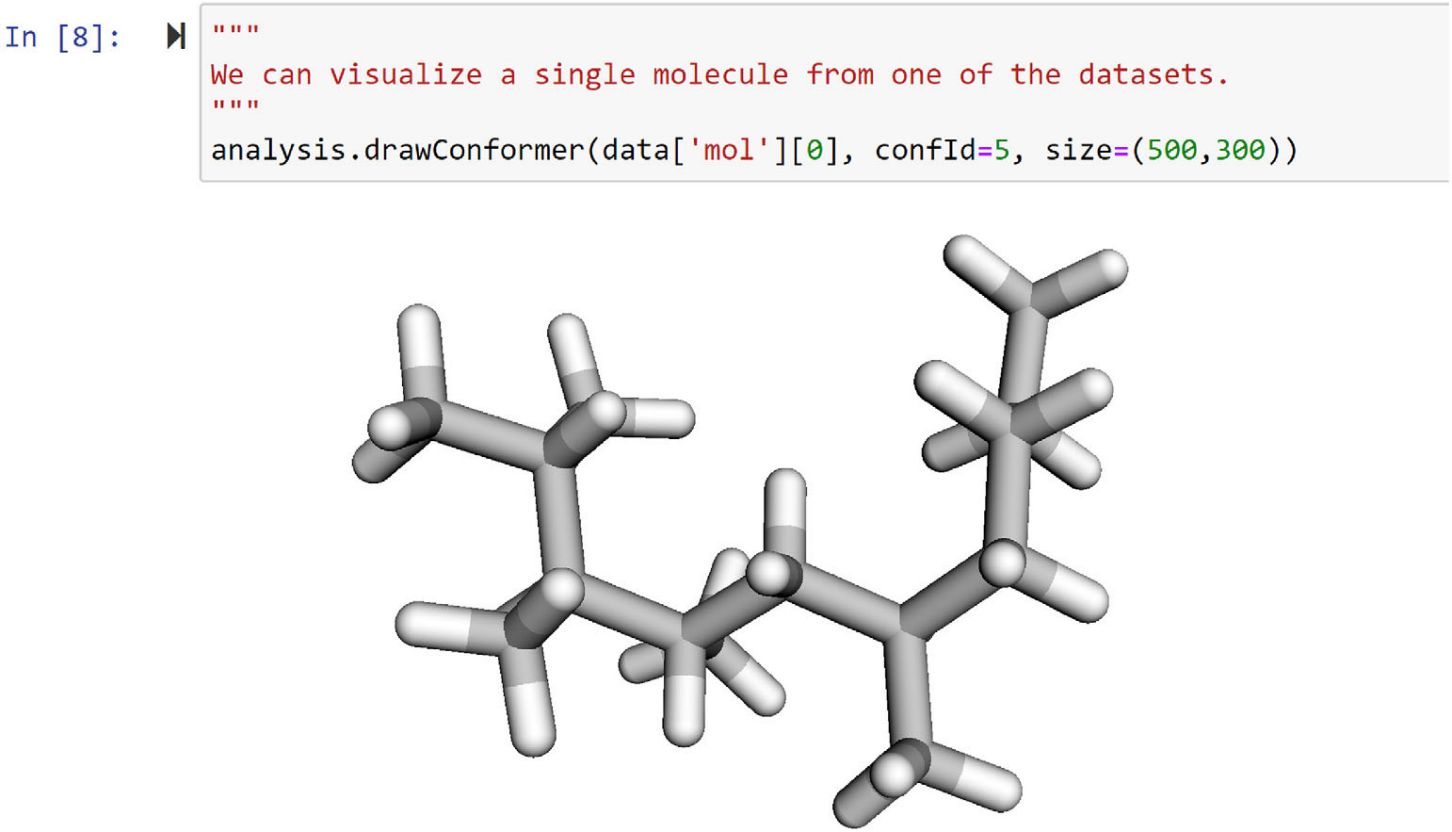
Example of using the toolkit to visualize a generated conformer in a Jupyter notebook

When evaluating a trained model, Conformer‐RL's EnvLogger module records RL environment information across a single RL environment interaction/episode, such as the conformers generated and conformer energies. EnvLogger supports saving the per‐episode data and each generated molecule conformer as a MOL file, so that the generated conformers can be used in further downstream analysis. It further contains an analysis toolkit for calculating and visualizing results in a Python notebook. The toolkit provides convenient methods for generating figures, charts, and interactive 3D visuals for molecule conformers. An example is shown in Figure 3.

## 3 EXAMPLE USAGE

We now demonstrate an example setup of how Conformer‐RL can be used to generate conformers for a lignin polymer containing eight monomers. Since the polymer is quite large and contains over 50 rotatable bonds, training an RL agent directly on the lignin polymer can take several days to achieve similar performance as SGMD. This is the case even when using a Nvidia Tesla V100 GPU and running 20 RL environments in parallel, since the number of possible actions scales exponentially with increasing number of rotatable bonds. One solution to this is to utilize curriculum learning. We will first train an RL agent on lignin polymers containing only 2–3 monomers. When the agent reached a performance threshold on the current polymer, it will be able to advanced and train on lignin polymers with sequentially larger number of monomers, up to six monomers total. The action space for these smaller lignin polymers are exponentially smaller than the action space for an eight monomer lignin, and our experiments indicate that less than 1 day of training is required for the RL agent to achieve similar performance using the same hardware. Thus, to utilize curriculum learning, we create an RL environment using Conformer‐RL by inputting a list of five lignin polymer structures, with the first structure containing two monomers, the second structure containing three monomers, and so on with the last structure containing six monomers. We then train a RL agent on this environment using the PPO algorithm.

**FIGURE 4:**
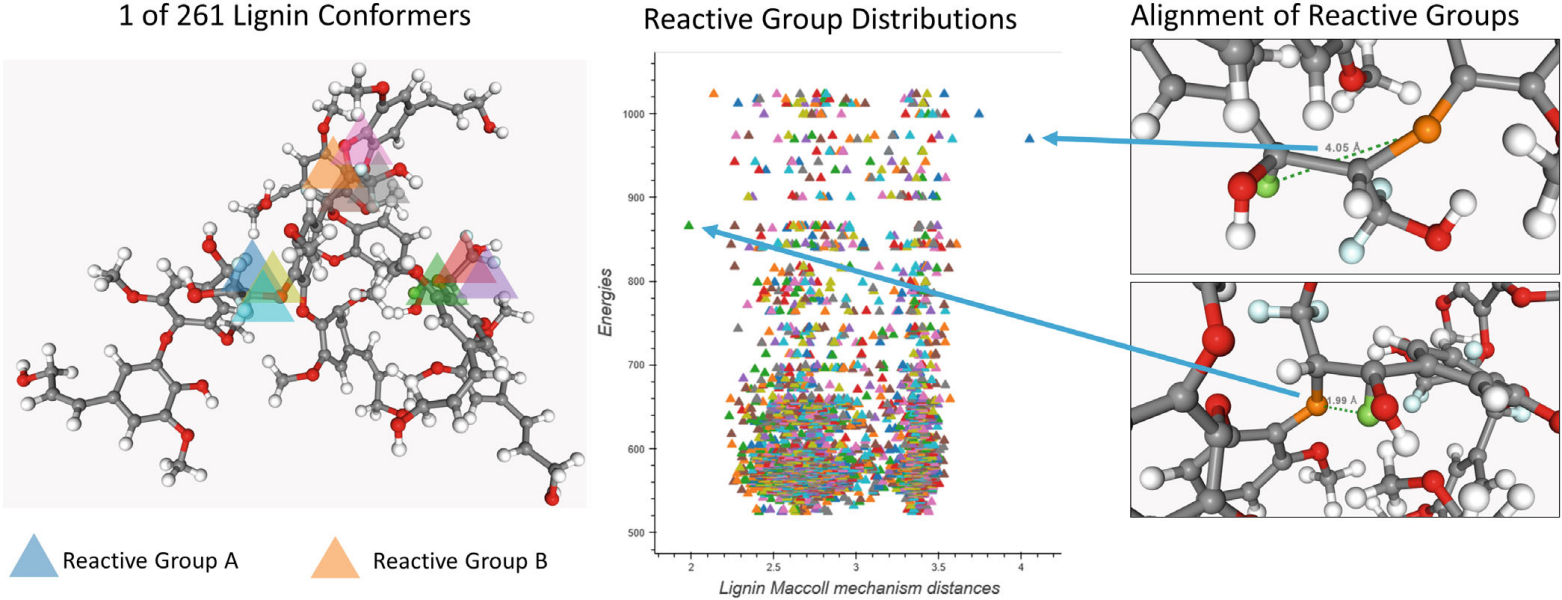
Analysis of conformers generated by conformer‐RL of lignin polymer with eight monomers. Highlighted are the proximity of reactive groups in the polymer, showing a distribution of interatomic distances for the Maccoll reaction mechanism

According to experiments reported by Gogineni et al.^9^ the model trained using this curriculum learning method is able to generalize well and successfully generate conformers for a lignin polymer with eight monomers, even outperforming SGMD. We can further use the saved .mol files dumped by the environment during evaluation to analyze the conformers generated for the 8‐lignin. An example is seen in Figure 4.

## 4 CONCLUSION

Conformer‐RL is a comprehensive library for training and testing deep RL agents in the conformer generation task. Conformer‐RL's modular interfaces can increase research reproducibility and stimulate discovery in conformer generation. We hope the availability of this library will bolster the computational chemistry community to engage advanced machine learning techniques for conformational sampling. Full documentation can be found at https://conformer-rl.readthedocs.io/en/latest/.

## Data Availability

Data sharing not applicable to this article as no datasets were generated or analyzed during the current study. The code for the software discussed in the paper is publicly available at https://github.com/ZimmermanGroup/conformer-rl.

## References

[1] D. Silver , T. Hubert , J. Schrittwieser , I. Antonoglou , M. Lai , A. Guez , M. Lanctot , L. Sifre , D. Kumaran , T. Graepel , T. Lillicrap , K. Simonyan , D. Hassabis , Science 2018, 362, 1140. 30523106 10.1126/science.aar6404

[2] O. Vinyals , I. Babuschkin , W. M. Czarnecki , M. Mathieu , A. Dudzik , J. Chung , D. H. Choi , R. Powell , T. Ewalds , P. Georgiev , J. Oh , D. Horgan , M. Kroiss, I. Danihelka, A. Huang, L. Sifre, T. Cai, J. P. Agapiou, M. Jaderberg, A. S. Vezhnevets, R. Leblond, T. Pohlen, V. Dalibard, D. Budden, Y. Sulsky, J. Molloy, T. L. Paine, C. Gulcehre, Z. Wang, T. Pfaff, Y. Wu, R. Ring, D. Yogatama, D. Wünsch, K. McKinney, O. Smith, T. Schaul, T. Lillicrap, K. Kavukcuoglu , D. Hassabis , C. Apps , D. Silver , Nature 2019, 575, 350. 31666705 10.1038/s41586-019-1724-z

[3] Y. Li , H. Kang , K. Ye , S. Yin , X. Li , Conference on Neural Information Processing Systems Deep Reinforcement Learning Workshop, Montréal, Canada, 2018. https://arxiv.org/abs/1812.00967

[4] Z. Zhou , X. Li , R. N. Zare , ACS Cent. Sci. 2017, 3, 1337. 29296675 10.1021/acscentsci.7b00492 PMC5746857

[5] G. Simm , R. Pinsler , J. M. Hernandez‐Lobato , in Proceedings of the 37th International Conference on Machine Learning, Vol. 119 (Eds: H. D. III , A. Singh ), PMLR, 2020, p. 8959 http://proceedings.mlr.press/v119/simm20b.html

[6] J.‐P. Ebejer , G. M. Morris , C. M. Deane , J. Chem. Inf. Model. 2012, 52, 1146. 22482737 10.1021/ci2004658

[7] J. C. Cole , O. Korb , P. McCabe , M. G. Read , R. Taylor , J. Chem. Inf. Model. 2018, 58, 615. 29425456 10.1021/acs.jcim.7b00697

[8] X. Wu , S. Wang , J Phys Chem B 1998, 102, 7238.

[9] T. Gogineni , Z. Xu , E. Punzalan , R. Jiang , J. Kammeraad , A. Tewari , P. Zimmerman , in Advances in Neural Information Processing Systems, Vol. 33 (Eds: H. Larochelle , M. Ranzato , R. Hadsell , M. F. Balcan , H. Lin ), Red Hook, NY: Curran Associates, 2020, p. 20142.

[10] E. Liang , R. Liaw , R. Nishihara , P. Moritz , R. Fox , K. Goldberg , J. Gonzalez , M. Jordan , I. Stoica , in Proceedings of the 35th International Conference on Machine Learning (Eds: J. Dy , A. Krause ), PMLR, 2018, p. 3053.

[11] G. Brockman , V. Cheung , L. Pettersson , J. Schneider , J. Schulman , J. Tang , W. Zaremba , Openai Gym 2016. https://arxiv.org/pdf/1606.01540.pdf

[12] A. Paszke , S. Gross , F. Massa , A. Lerer , J. Bradbury , G. Chanan , T. Killeen , Z. Lin , N. Gimelshein , L. Antiga , A. Desmaison , A. Kopf , E. Yang , Z. DeVito , M. Raison , A. Tejani , S. Chilamkurthy , B. Steiner , L. Fang , J. Bai , S. Chintala , in Advances in Neural Information Processing Systems 32 (Eds: H. Wallach , H. Larochelle , A. Beygelzimer , F. D.'. Alché‐Buc , E. Fox , R. Garnett ), Red Hook, NY: Curran Associates, 2019, p. 8024 http://papers.neurips.cc/paper/9015-pytorch-an-imperative-style-high-performance-deep-learning-library.pdf

[13] G. Landrum , rdkit , 2016. https://github.com/rdkit/rdkit/releases/tag/Release_2016_09_4

[14] N. M. O'Boyle , T. Vandermeersch , C. J. Flynn , A. R. Maguire , G. R. Hutchison , J Cheminform 2011, 3, 1. 21410983 10.1186/1758-2946-3-8 PMC3073927

[15] N. M. O'Boyle , M. Banck , C. A. James , C. Morley , T. Vandermeersch , G. R. Hutchison , Aust. J. Chem. 2011, 3, 33. 10.1186/1758-2946-3-33 PMC3198950 21982300

[16] L. Turcani , A. Tarzia , F. T. Szczypiński , K. E. Jelfs , J Chem Phys 2021, 154, 214102. 34240979 10.1063/5.0049708

[17] L. T. Steven Bennett , A. Tarzia , stko , 2021. https://github.com/JelfsMaterialsGroup/stko

[18] M. J. Orella , T. Z. H. Gani , J. V. Vermaas , M. L. Stone , E. M. Anderson , G. T. Beckham , F. R. Brushett , Y. Román‐Leshkov , ACS Sustainable Chem. Eng. 2019, 7, 18313.

[19] A. L. Dewyer , A. J. Argüelles , P. M. Zimmerman , WIREs Comput Mol Sci 2018, 8, e1354.

[20] T. A. Halgren , R. B. Nachbar , J. Comput. Chem. 1996, 17, 587.

[21] T. Schulz‐Gasch , C. Schärfer , W. Guba , M. Rarey , J. Chem. Inf. Model. 2012, 52, 1499. 22670896 10.1021/ci2002318

[22] Y. Wu , E. Mansimov , R. B. Grosse , S. Liao , J. Ba , in Advances in Neural Information Processing Systems, Vol. 30 (Eds: I. Guyon , U. V. Luxburg , S. Bengio , H. Wallach , R. Fergus , S. Vishwanathan , R. Garnett ), Red Hook, NY: Curran Associates, 2017. https://proceedings.neurips.cc/paper/2017/file/361440528766bbaaaa1901845cf4152b-Paper.pdf

[23] J. Schulman , F. Wolski , P. Dhariwal , A. Radford , O. Klimov , Proximal Policy Optim Algorith 2017, 1707, 06347.

[24] M. Fey , J. E. Lenssen , ICLR Workshop on Representation Learning on Graphs and Manifolds, New Orleans, USA, 2019.

[25] S. Narvekar , J. Sinapov , M. Leonetti , P. Stone , *Proceedings of the 15th International Conference on Autonomous Agents and Multiagent Systems (AAMAS 2016)*, Singapore, 2016.

[26] C. Cai , S. Wang , Y. Xu , W. Zhang , K. Tang , Q. Ouyang , L. Lai , J. Pei , J. Med. Chem. 2020, 63, 8683. 32672961 10.1021/acs.jmedchem.9b02147

[27] M. Abadi , A. Agarwal , P. Barham , E. Brevdo , Z. Chen , C. Citro , G. S. Corrado , A. Davis , J. Dean , M. Devin , S. Ghemawat , I. Goodfellow , A. Harp , G. Irving , M. Isard , Y. Jia , R. Jozefowicz , L. Kaiser , M. Kudlur , J. Levenberg , D. Mane , R. Monga , S. Moore , D. Murray , C. Olah , M. Schuster , J. Shlens , B. Steiner , I. Sutskever , K. Talwar , P. Tucker , V. Vanhoucke , V. Vasudevan , F. Viegas , O. Vinyals , P. Warden , M. Wattenberg , M. Wicke , Y. Yu , X. Zheng , TensorFlow (Version v2.8.2), 2015. https://static.googleusercontent.com/media/research.google.com/en//pubs/archive/45166.pdf

